# Preoperative OCT Biomarkers as Predictors of Postoperative Functional Outcome Assessed by Microperimetry After Inverted ILM Flap Surgery

**DOI:** 10.3390/diagnostics16121919

**Published:** 2026-06-20

**Authors:** Ovidiu Samoilă, Anca Mădălina Sere, Lăcrămioara Samoilă, Daniel-Corneliu Leucuța

**Affiliations:** 1 Department of Ophthalmology, “Iuliu Hațieganu” University of Medicine and Pharmacy, 400012 Cluj-Napoca, Romania; ciprian.samoila@umfcluj.ro (O.S.); sere.anca16@gmail.com (A.M.S.); 2 Department of Physiology, “Iuliu Hațieganu” University of Medicine and Pharmacy, 400012 Cluj-Napoca, Romania; 3 Department of Medical Informatics and Biostatistics, “Iuliu Hațieganu” University of Medicine and Pharmacy, 400012 Cluj-Napoca, Romania; dleucuta@umfcluj.ro

**Keywords:** macular hole, inverted flap surgery, pars plana vitrectomy, ocular coherence tomography, microperimetry, prognostic factors, machine learning

## Abstract

**Background/Objectives**: A macular hole represents a significant surgical condition in an increasingly aging population. Advances in surgical techniques, particularly pars plana vitrectomy with inverted internal limiting membrane (ILM) flap, have established high anatomical closure rates exceeding 90%. The prognostic factors influencing visual recovery remain incompletely understood, and it is unclear which patients can be expected to achieve optimal functional outcomes. **Methods**: This retrospective longitudinal study included 35 eyes of 32 patients followed for 3–12 months. Preoperative OCT parameters (minimum linear diameter, basal diameter, and hole height) and derived indices were correlated with functional outcomes, including best-corrected visual acuity (BCVA) and microperimetry, stratified as central macular sensitivity (CMS) and sensitivity at 4° and 20°. Postoperative ellipsoid zone (EZ) and external limiting membrane (ELM) integrity were also analyzed. Predictive performance was assessed using root mean square error (RMSE) and coefficient of determination (R^2^). A linear regression model based on BCVA served as baseline, while Extreme Gradient Boosting (XGBoost) models incorporating OCT features were developed. Feature importance was evaluated using Shapley Additive Explanations (SHAP). **Results**: Overall closure rate was 100%, including 91.4% Type 1 and 8.6% Type 2 closure. Models incorporating OCT parameters outperformed BCVA-based models (lower RMSE, and higher R^2^). Minimum linear diameter and hole height were the strongest predictors of postoperative outcomes. Microperimetry detected functional improvement beyond BCVA and correlated with EZ and ELM restoration. **Conclusions**: Preoperative macular hole morphology represents a key determinant of postoperative functional recovery. These structural parameters provide meaningful prognostic value beyond visual acuity alone, supporting the role of combined OCT and microperimetric assessment in predicting surgical outcomes.

## 1. Introduction

Senescence is increasingly affecting the global population, with significant implications for ocular health. The retina, characterized by one of the highest metabolic demands in the human body, is particularly susceptible to degenerative changes, especially at the macular level. Macular hole (MH) represents one such degenerative condition, and full-thickness macular hole (FTMH) requires highly precise surgical intervention due to the delicate nature of retinal tissue.

Pars plana vitrectomy has become a well-established procedure for MH repair, achieving high success rates, particularly with recent advances in macular peeling and the inverted internal limiting membrane (ILM) flap technique. Anatomical closure is reported in 90–100% of cases. Early studies demonstrated that the inverted ILM flap improves anatomical outcomes in large macular holes (>400 µm) compared to conventional ILM peeling [1]. However, functional outcomes do not always parallel anatomical success and may remain suboptimal for both patients and surgeons.

The prognostic factors influencing visual recovery remain incompletely understood, and it is unclear which patients can be expected to achieve optimal functional outcomes in terms of visual acuity and visual field. While the inverted flap technique was initially developed for large MHs, more recent studies suggest no clear advantage in closure rate or visual acuity outcomes for small MHs [2,3]. Notably, most of these studies have focused primarily on visual acuity, whereas more sensitive methods of functional assessment, such as microperimetry, have been rarely employed, and in small cohorts [4,5,6,7,8]. Visual acuity provides only a limited representation of macular function and frequently fails to reflect subtle functional changes during retinal recovery. Furthermore, previous studies have generally evaluated individual optical coherence tomography (OCT) biomarkers using conventional statistical approaches, whereas the relative prognostic contribution of multiple anatomical parameters has not been systematically explored using explainable machine-learning methods. Therefore, it remains uncertain which preoperative morphological characteristics are most important for predicting postoperative retinal sensitivity following inverted ILM flap surgery.

The aim of the present study was to determine which preoperative OCT-derived morphological characteristics are most strongly associated with postoperative retinal function assessed by microperimetry after inverted ILM flap surgery. In addition, we sought to evaluate the relative prognostic importance of individual OCT biomarkers using explainable machine-learning techniques and to compare their predictive value with conventional visual acuity measurements. Given the scarcity of previous reports and the application of robust statistical methods, our study aimed to further expand current knowledge on functional rehabilitation after successful macular hole closure.

## 2. Materials and Methods

### 2.1. Study Design and Setting

This study was designed as a retrospective longitudinal observation study of patients having full thickness macular hole and pars plana vitrectomy with an inverted ILM flap technique. We reviewed the medical records of patients who underwent vitrectomy between 2024 and 2025. All surgeries were performed in a single center (The Ophthalmology Clinic, Cluj-Napoca, a tertiary hospital), by one surgeon (O.S.).

### 2.2. Participants

Inclusion criteria were: diagnosis of FTMH, inverted ILM flap technique, patients with full medical records (in the electronic archive), availability of high-quality preoperative and postoperative optical coherence tomography (OCT) imaging, visual acuity before and after the surgery, microperimetry, full video files from the surgery (to confirm the procedure), and minimum follow-up for 3 months, including OCT and microperimetry assessments. The rate of success and functional and structural integrity were noted. The first recorded postoperative examination was performed approximately 1 month after surgery and was designated as Follow-up 1. Follow-up 3 corresponded to the examination performed approximately 3 months postoperatively. The final follow-up was defined as the last available postoperative assessment, performed between 3 and 12 months after surgery. Longitudinal analyses were based on comparisons among these predefined postoperative time points.

Exclusion criteria were: patients with incomplete data (visual acuity, OCT, microperimetry), poor image quality precluding reliable measurement, incomplete follow-ups, previous vitreoretinal surgeries, and lamellar macular holes. In the end, 35 eyes of 32 patients were included.

This research followed internationally accepted ethical guidelines as outlined in the Declaration of Helsinki, and all participants gave written informed consent for medical research and teaching at the time of the surgery. The study was approved by the ethics committee of the “Iuliu Hațieganu” University of Medicine and Pharmacy Cluj-Napoca (No. 104/14 April 2025). All patient data was anonymized prior to analysis.

### 2.3. Surgery Technique

All surgeries were performed using standard 23-gauge pars plana vitrectomy. Following core vitrectomy and posterior hyaloid detachment, the internal limiting membrane was peeled in a circular fashion around the macular hole. Triamcinolone was also used for vitreous staining and for ILM indirect staining (mechanical visualization). An inverted ILM flap was created and positioned over the hole (either covering the defect, or “tucked in”, depending on the hole dimension). Fluid–air exchange was performed, followed by gas tamponade (C2F6). Patients were instructed to maintain a prone position postoperatively for 4 h and to avoid a face-up position for the next 72 h.

### 2.4. OCT Imaging and Analysis

**Figure 1:**
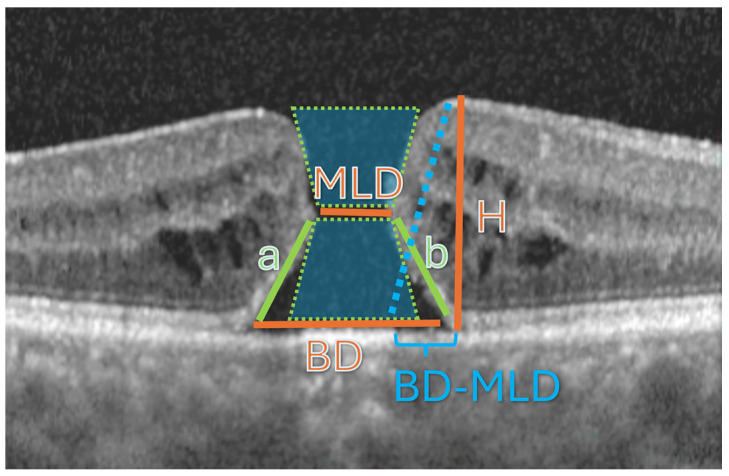
Basic OCT parameters of macular holes. MLD (minimum linear diameter), BD (basal diameter), and H (maximum height). The hole is approximated to a symmetric hourglass (and a = b) to calculate HFF (hole form factor).

Spectral-domain OCT (Heidelberg Engineering, Heidelberg, Germany) was performed preoperatively and at multiple postoperative visits (1, 3, 6, and up to 12 months). The following parameters were measured from preoperative OCT images: Minimum linear diameter (MLD), Base diameter (BD), and Macular hole height (H) (Figure 1).

In addition to the primary anatomical measurements, several previously described macular hole indices were calculated, allowing a better geometric characterization of the hole. The Macular Hole Index (MHI) was calculated as H/BD, the Diameter Hole Index (DHI) as MLD/BD, and the Tractional Hole Index (THI) as H/MLD, according to the methods originally described by Kusuhara et al. [9] and Ruiz-Moreno et al. [10] A variation of the hole form factor (HFF) was calculated based on BD, MLD, and H:

HFF = 2 × sqrt(H^2^ + (BD−MLD)^2^)/BD.

Macular holes were classified according to both the Gass staging system and the International Vitreomacular Traction Study (IVTS) group classification [11]. The Gass classification was used to describe the stage of disease progression (Stages 2–4), based on the clinical and anatomical characteristics of the macular hole. In parallel, the IVTS classification was applied to categorize macular holes according to their minimum linear diameter measured on OCT, as small (<250 µm), medium (250–400 µm), or large (>400 µm). The use of both classification systems allowed characterization of the cohort from both a clinical and morphological perspective and facilitated comparison with previous studies.

Postoperative OCT evaluation included: type of macular hole closure, integrity of the external limiting membrane (ELM), and restoration of the ellipsoid zone (EZ).

Postoperative ELM and EZ status were classified as continuous, discontinuous, or absent/not identifiable. A layer was considered continuous when a hyperreflective band could be followed across the foveal region without interruption. It was classified as discontinuous when a visible interruption persisted within the foveal area, and absent/not identifiable when the corresponding band could not be reliably detected.

The extent of ELM and EZ disruption was measured manually on the horizontal OCT B-scan passing through the foveal center using the built-in Heidelberg caliper tool. Disruption diameter was defined as the linear distance between the nearest visible edges of the intact band and recorded in micrometers. When the layer was continuous, disruption diameter was recorded as 0 µm.

### 2.5. Microperimetry Assessment

Microperimetry (Nidek MP-3, NIDEK Co., Ltd., Gamagori, Japan) was performed using a standardized surgical protocol. Functional parameters recorded included mean retinal sensitivity (MS), and central macular sensitivity (CMS). Precise auto-tracking and auto-alignment allowed for the follow-up test to be performed on the same area, with the same parameters as the previous test. The MP-3 protocol evaluated 33 retinal loci distributed within the central 20°. Measured points were located at 0, 2, 6 and 10 degrees from the center. Only reliable examinations (based on fixation losses and tracking stability) were included in the analysis. Central macular sensitivity (CMS) corresponded to the sensitivity of the central fixation point. Mean sensitivity within the central 4° (MS 4°) was calculated as the average sensitivity of the 9 central test points, whereas mean sensitivity within the central 20° (MS 20°) represented the average of all 33 test points. The broader 20° analysis was included to differentiate localized foveal recovery from more generalized changes in macular sensitivity. The 33-point protocol largely overlaps with the ETDRS grid on OCT. This allowed us to correlate retinal sensitivity measurements with anatomical changes observed within the same central macular region.

### 2.6. Outcome Measurements

The primary outcome was postoperative functional status, assessed by microperimetric mean sensitivity and best corrected visual acuity (BCVA). Secondary outcomes included correlations between preoperative OCT biomarkers and postoperative structural and functional parameters. Visual function was evaluated using the decimal system, while for the statistical analysis, this was converted into LogMAR. Microperimetric measurements were stratified into average MS for the center (CMS), central 4° (9 points), 12° (17 points), and 20° (33 points).

### 2.7. Statistical Analysis

Statistical analysis was performed using R software 4.6.0 for Windows (R Foundation for Statistical Computing, Vienna, Austria) and SPSS (IBM SPSS v31). Continuous variables were expressed as mean ± standard deviation or median (interquartile range IQR 25–75%), depending on data distribution (verified with Shapiro–Wilk test and Kolmogorov–Smirnov).

Correlations between variables were assessed using Spearman’s rank correlation coefficient. Univariate analyses were performed to identify potential predictors of postoperative functional outcomes. Variables with significant associations were included in multivariate regression models, with the number of predictors limited according to sample size considerations.

Predictive performance of the different prognostic models was evaluated using root mean square error (RMSE) and the coefficient of determination (R^2^). A linear regression model was used for preoperative visual acuity alone, while XGBoost models were employed to assess the prognostic contribution of preoperative OCT-derived measurements and indices. Feature contributions were assessed using SHAP (Shapley Additive Explanations), which quantifies the impact of each predictor on model outputs. Mean absolute SHAP values were calculated to quantify global feature importance across all observations. Additionally, SHAP dependence plots were generated to evaluate the relationship between individual predictors and their contribution to model predictions. For these analyses, SHAP values were computed for each observation, and locally weighted smoothing was applied to visualize trends in dependence plots. Positive and negative SHAP values were interpreted relative to the model baseline prediction.

A *p*-value < 0.05 was considered statistically significant.

## 3. Results

Pars plana vitrectomy with inverted ILM flap was performed on 35 eyes of 32 patients in the interval 2024–2025. Anatomical closure was achieved in all 35 eyes. Type 1 closure was observed in 32 eyes (91.4%), whereas 3 eyes (8.6%) developed Type 2 closure, with delayed retinal remodeling during follow-up.

### 3.1. Population

Demographic distribution, visual acuity before surgery, and macular hole characteristics on OCT are displayed in Table 1. Average follow-up was 171.1 days.

### 3.2. Outcome

**Figure 2:**
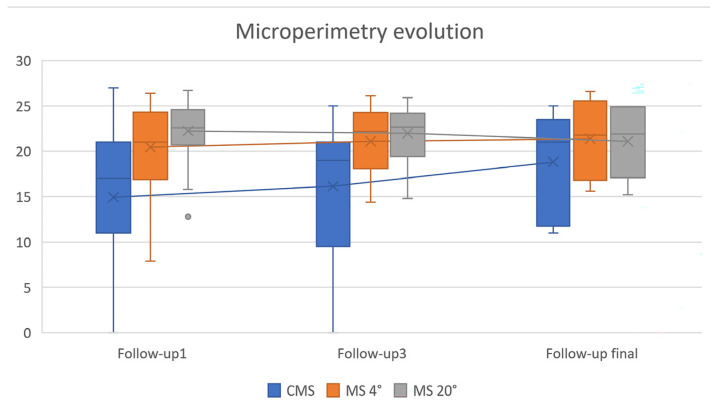
Microperimetry results as boxplots (median, interquartile range) at 3 postoperative follow-ups (1 month, 3 months, and final). CMS, central macular sensitivity, MS 4°, microperimetry in the central 4 degrees, and MS 20°, microperimetry in the central 20 degrees.

**Figure 3:**
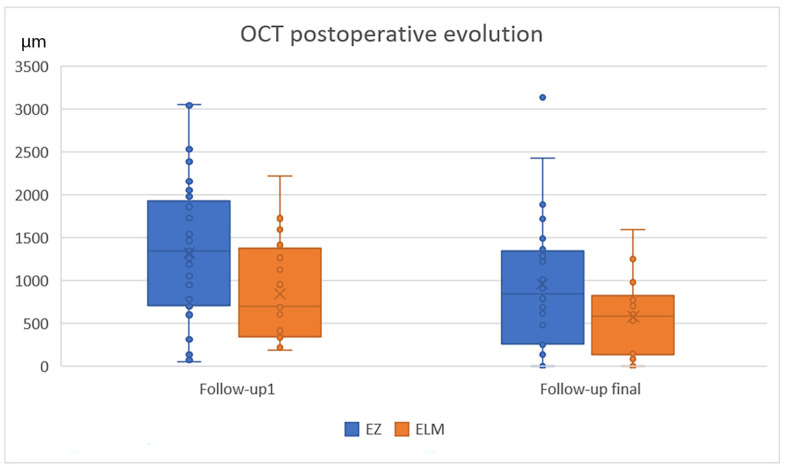
OCT postoperative evolution as boxplots (median, interquartile range), regarding EZ and ELM disruption reduction (from 1 month postoperative to the end of follow-up) ELM, External Limiting Membrane; EZ, Ellipsoid Zone. Only eyes with measurable ELM/EZ defects were included.

The evolution of retinal function (visual acuity—BCVA in LogMAR units, and microperimetry) and structure (OCT—integrity of EZ and ELM) are presented in Table 2 and Figure 2 and Figure 3.

There was an improvement in BCVA, accompanied by a progressive reduction in disruption of the EZ and ELM.

BCVA was significantly improved following the surgery at 1 month (*p* < 0.0001, *t*-test), remaining stable afterwards. Postoperative CMS, MS 4°, and MS 20° were significantly lower than the fellow eye, at 1 month (*t*-test, *p* = 0.01, *p* = 0.001, and *p* = 0.008, respectively). However, by the final follow-up, no significant differences were observed between postoperative values of the treated eye and the baseline microperimetry measurements of the fellow (healthy) eye for CMS (*p* = 0.518), MS 4° (*p* = 0.472), and MS 20° (*p* = 0.166).

Microperimetry demonstrated variability, particularly in CMS and central 4° (MS 4°), with an overall trend toward improvement (CMS increased from 14.94 to 16.50 dB; MS 4° from 20.44 to 21.36 dB), while MS 20° remained relatively stable across all postoperative visits. At each follow-up (1 month, 3 months, and final—variable), CMS showed progressive improvement (Figure 2), reaching statistical significance between 1 and 3 months (*t*-test, *p* = 0.04), and remaining stable thereafter. Although MS 4° improved over the same interval, this change did not reach statistical significance. Furthermore, 17 eyes (48%) presented lens opacities, without surgical indication for lens exchange (the impact on BCVA was considered negligible). This may have influenced, however, to a variable degree, functional results in the long-term. Contrary to BCVA, microperimetry constantly improved after first follow-up.

ELM integrity improved during follow-up, with continuous ELM observed in 66% of cases, compared to 31% at 1 month postoperatively (Wilcoxon signed-rank test, *p* < 0.001). In the remaining 34%, the ELM persisted as discontinuous or absent. A similar trend was noted for the EZ, continuous EZ increasing from 6% at 1 month to 40% at final follow-up (Wilcoxon signed-rank test, *p* < 0.001). The lengths of both ELM and EZ disruption, as measured on OCT, decreased significantly over time (170.04 µm reduction, *p* = 0.014, and 229.89 µm reduction, *p* = 0.003, respectively; *t*-test) (Figure 3).

### 3.3. Associations

#### 3.3.1. Correlations

Table 3 illustrates the associations between visual acuity, microperimetry, and OCT findings, both preoperative (MH parameters) and postoperative (ELM and EZ).

ELM status successfully predicted BCVA (at each follow-up) and retinal sensitivity, in the center—CMS, 4°, but not 20°. EZ status was significant for the prediction of BCVA and the central macular sensitivity, but not retinal sensitivity in the central 4°, or 20°. The amount of disruption of EZ was, however, significant for CMS and MS 4°, at all follow-up visits.

The preoperative OCT parameters were well correlated with postoperative function. The strongest correlations were observed regarding BD, MLD, and HFF, with all functional parameters (BCVA, CMS, MS 4°, and MS 20°). This association was further explored in the Prognostic models.

#### 3.3.2. Prognostic Models

To assess the incremental prognostic value of preoperative OCT measurements beyond visual acuity for postoperative central retinal sensitivity, we built several models (Table 4). Preoperative visual acuity alone showed limited ability to predict postoperative central 4-degree microperimetric sensitivity (R^2^ = 0.04). OCT-derived measurements provided substantial prognostic information when considered independently, with raw anatomical dimensions outperforming derived indices. The strongest predictive performance was achieved when preoperative visual acuity was combined with raw OCT measurements (R^2^ = 0.85), indicating that absolute macular hole geometry provides complementary prognostic information beyond baseline function. Models incorporating both raw measurements and derived indices performed less well, suggesting redundancy between representations.

To further explore the relative importance of variables (as measured by gain) in predicting central retinal sensitivity, we used the best model that included BCVA and raw OCT-derived parameters (Table 5). Hole height showed the most important contribution to the prediction ability of the model, followed by minimum linear diameter and basal diameter, which contributed to a similar extent. In contrast, preoperative visual acuity showed a clearly lower Gain. Overall, these findings suggest that absolute macular hole dimensions, particularly vertical height and horizontal extent, are the most informative preoperative predictors of postoperative central 4-degree microperimetric sensitivity within the model.

**Figure 4:**
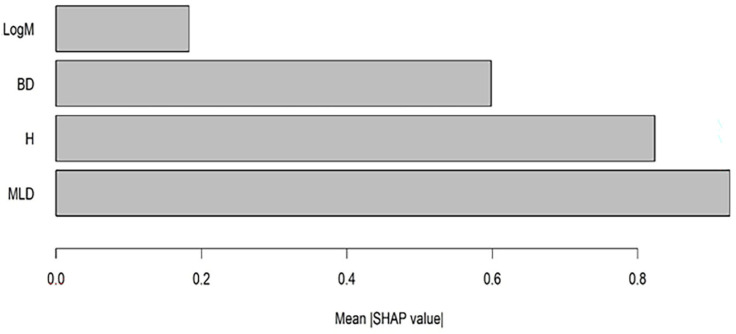
SHAP feature importance for XGBoost predicting MS 4° based on preoperative BCVA, BD, MLD, and H. Higher values indicate greater importance. Bars represent mean absolute SHAP values, indicating the average contribution of each predictor to model predictions across all patients.

**Figure 5:**
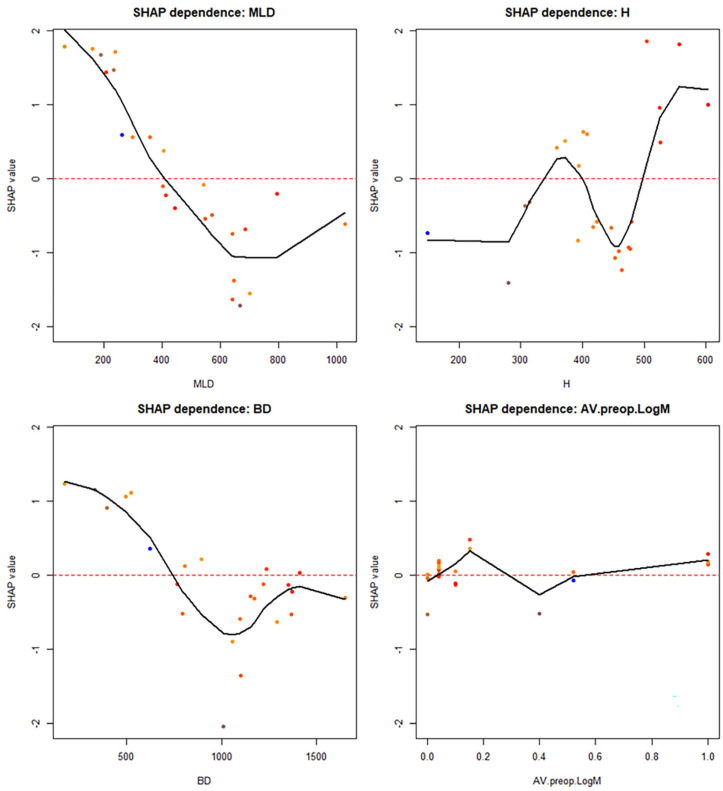
Each panel shows the relationship between an individual predictor and its SHAP value, representing the contribution of that variable to the predicted postoperative central 4-degree microperimetric sensitivity. Points represent individual patients, and the solid line indicates a locally smoothed trend. Positive SHAP values indicate higher predicted sensitivity relative to the model baseline, while negative values indicate lower predicted sensitivity. The plots illustrate nonlinear and threshold-dependent effects of macular hole geometry, whereas preoperative visual acuity shows a comparatively modest influence.

A complementary exploration of the relative importance of variables, but in the sense of how much one variable modifies the predicted values, is presented in Figure 4 and Figure 5, which uses SHAP. The results are similar in the sense that the absolute macular hole dimensions were more important than the preoperative visual acuity. However, the minimum linear diameter was the most influential parameter, followed by hole height and basal diameter. Furthermore, these relationships were nonlinear.

Taken together, these analyses identify hole height and minimum linear diameter as the most important variables in terms of relative importance for prediction. Derived parameters may hold less importance.

## 4. Discussion

This longitudinal cohort study found that pars plana vitrectomy with the inverted ILM flap technique resulted in excellent anatomical and functional outcomes. While previous studies have primarily focused on visual acuity outcomes, we evaluated retinal sensitivity using microperimetry and explored the relative prognostic importance of multiple OCT-derived biomarkers using explainable machine-learning techniques.

Visual function improved significantly, as observed by improvements in BCVA and central retinal sensitivity, especially within the central macula (CMS and MS 4°), while MS 20° remained relatively stable. Also, there was an improvement in structural recovery, with significant restoration of both ELM and EZ integrity over time. Correlation analyses confirmed a relationship between structure and function, with ELM and EZ status associated with BCVA and central sensitivity, and the EZ disruption showing associations with central functional parameters. Preoperative OCT characteristics, especially BD, MLD, and HFF, were strongly correlated with postoperative outcomes, showing the importance of baseline macular hole geometry.

Furthermore, prognostic modeling showed that preoperative OCT-derived parameters provide predictive value beyond visual acuity. While baseline BCVA showed limited predictive ability, models incorporating absolute macular hole dimensions achieved high explanatory power, especially when combined with BCVA. Results consistently identified hole geometry, especially minimum linear diameter and hole height, as the most influential predictors, in determining postoperative functional recovery.

Pars plana vitrectomy with ILM peeling remains the standard treatment for full-thickness macular holes. While ILM peeling has demonstrated high closure rates for small (≤250 μm) and medium (251–400 μm) MHs, many authors have reported significantly lower closure rates for MHs larger than 400 μm [11,12,13]. The introduction of the inverted ILM flap technique by Michalewska et al. [1] significantly improved anatomical outcomes in large macular holes, with closure rates approaching 90–100% in most contemporary series. Our findings are consistent with these reports, achieving anatomical closure in all eyes and Type 1 closure in 91.4% of cases.

Several modifications of the inverted ILM flap technique have subsequently been proposed; however, most studies report comparable anatomical outcomes among the different variants. Therefore, the present study focused primarily on postoperative functional recovery and its relationship with preoperative macular hole morphology.

The precise mechanism underlying the success of the inverted ILM flap technique remains incompletely understood. However, previous OCT studies have consistently demonstrated progressive restoration of the outer retinal layers following surgery, typically beginning with recovery of the ELM and followed by gradual reconstruction of the EZ. Following the inverted ILM flap technique, retinal tissue begins to regenerate from the external limiting membrane (ELM), with subsequent reconstruction of the ellipsoid layer (EZ) and interdigitation zone (IZ) over the ensuing months [14,15]. In our cohort, this regenerative process was reflected by a significant improvement in ELM integrity over time, with complete restoration increasing from 31% at 1 month postoperatively to 66% at final follow-up. A similar, although delayed, trend was observed for the EZ, where restoration increased from 6% to 40%, supporting the concept that ELM recovery precedes and possibly facilitates photoreceptor layer reconstruction.

Moreover, complete ELM restoration has been identified as a key prognostic factor for best-corrected visual acuity (BCVA) improvement [16]. Our results align with these observations, as ELM integrity was consistently associated with both BCVA and central macular sensitivity at all follow-up visits, reinforcing its role as a reliable indicator of functional recovery. Furthermore, the gradual disappearance of glial tissue appears to promote both the structural and functional recovery of photoreceptors [15]. Since the microstructural regeneration of retinal layers can continue for up to 2 years, it is essential to inform patients about the prolonged nature of functional recovery [17].

For small and medium MHs, there is currently no strong consensus on the optimal surgical technique. Our study included 63% eyes with large macular holes, 14% medium holes, and 23% small holes. There was no difference in closure success, but functional outcomes were highly dependent to macular hole size. Chou et al. [18] conducted a retrospective study focusing on MHs < 400 μm, comparing the outcomes of the inverted ILM flap technique and conventional ILM peeling. While both procedures successfully achieved macular hole closure and improved vision, the inverted ILM flap method was associated with a significantly faster recovery of visual function. Furthermore, patients in this group showed less foveal gliosis at 1 month, greater restoration of the ELM at 3 months, and enhanced IZ restoration at 3, 6, and 12 months postoperatively. Ventre et al. used microperimetry to evaluate functional outcomes and reported a better final mean sensitivity (MS) in eyes treated with conventional ILM peeling [7]. In 2024, a meta-analysis of over 600 eyes found no statistically significant differences in MH closure rates between the inverted ILM flap technique (99.4%) and conventional ILM peeling (96.2%) for small and medium macular holes. Furthermore, both techniques showed comparable outcomes in VA recovery, retinal sensitivity, and the restoration of the ellipsoid zone (EZ) and ELM [19].

OCT-derived biomarkers have become increasingly important for predicting functional recovery after macular hole surgery. Previous studies have identified minimum linear diameter, basal diameter, and several derived indices as potential predictors of postoperative visual outcomes. Our findings support these observations and further demonstrate their association with postoperative retinal sensitivity assessed by microperimetry.

Regarding the quantification of visual function in macular diseases, standard methods such as BCVA show a weak correlation between morphological and functional aspects [20]. Studies that have quantified visual function using microperimetry support its superiority over BCVA, demonstrating that retinal sensitivity changes even when VA remains stable [21,22]. The MP-3 microperimeter (Nidek Technologies, Padua, Italy) offers a novel approach for objectively and quantitatively measuring retinal sensitivity (MS), with significant potential for macular disease management [23]. Unlike BCVA, which measures overall central retinal performance, microperimetry targets specific central retinal areas and combines MS assessments with fundus imaging. This makes it especially useful for correlating MS with OCT structural changes and understanding visual recovery after vitreoretinal interface surgery [24]. In our study, microperimetric sensitivity improved after surgery and continued to increase throughout the follow-up period. In contrast, BCVA showed a rapid postoperative improvement but remained largely stable thereafter, suggesting an early plateau in conventional visual acuity outcomes. The evolution of microperimetry closely paralleled the structural recovery observed in OCT, particularly the restoration of the ellipsoid zone (EZ) and external limiting membrane (ELM). As these outer retinal layers progressively reconstituted, a corresponding improvement in retinal sensitivity was detected, even in cases where BCVA changes were minimal. These findings support the notion that microperimetry is a more sensitive functional biomarker than BCVA, capable of capturing subtle changes in macular function. While BCVA reflects central, high-contrast visual performance, microperimetry provides a more nuanced assessment of retinal function, better correlating with the gradual anatomical recovery of photoreceptors.

### 4.1. Limitations

The retrospective design limits control over potential confounding factors. However, multiple variables were incorporated into several models to mitigate this effect. Despite these adjustments, residual confounding remains an inherent limitation of observational studies. The single-center, single-surgeon setting, while ensuring consistency, may limit the generalizability of the findings. Microperimetry, although precise, remains dependent on patient performance and test reliability, which may introduce variability despite quality control measures.

Symptom duration could not be reliably retrieved from all medical records because of the retrospective design and was therefore not included in the analysis. Since chronicity is a recognized predictor of functional outcome after macular hole surgery, this represents an additional limitation of the present study. Nearly half of the eyes presented mild lens opacities that did not warrant cataract surgery. Although considered clinically insignificant, a small influence on long-term functional outcomes cannot be completely excluded.

The relatively small sample size represents an important limitation, particularly for predictive modeling, but this study is an exploratory one, so the methods are appropriate. While the machine-learning analyses provided useful exploratory insights regarding the relative prognostic importance of preoperative OCT biomarkers, the possibility of model overfitting cannot be completely excluded. Therefore, the predictive performance metrics reported in this study should be interpreted cautiously. The models should be regarded as hypothesis-generating rather than clinically deployable prediction tools, and external validation in larger independent cohorts will be necessary before routine clinical implementation.

### 4.2. Strengths

This study is among the relatively few to evaluate postoperative outcomes of the inverted flap technique using a combined structural–functional approach with both OCT and microperimetry, and even fewer studies have integrated these assessments with explainable machine-learning methods. The microperimetric data were further stratified into concentric regions (central, 4°, and 20°), enabling a more precise topographic correlation between retinal sensitivity and outer retinal layer integrity. One study strength is its longitudinal design with standardized follow-up. Surgical uniformity was ensured by a single surgeon, reducing intervention variability. The integration of high-resolution OCT biomarkers with microperimetry provides a comprehensive assessment of structure and function relationships, offering greater sensitivity than visual acuity alone.

Another important strength of the present study is the integration of machine learning techniques to investigate the relationship between preoperative macular hole morphology and postoperative functional outcomes. Although previous studies evaluating inverted ILM flap surgery have primarily relied on conventional statistical analyses, few have attempted to model the complex and potentially nonlinear interactions between OCT biomarkers and visual recovery. By employing XGBoost algorithms and SHAP-based explainability analysis, our study moved beyond simple correlation testing and allowed identification of the relative contribution of individual anatomical parameters to postoperative function. This approach consistently highlighted minimum linear diameter as the most influential predictor, followed by hole height and basal diameter, while also revealing nonlinear relationships that would likely remain undetected using traditional regression methods alone. To the best of our knowledge, this is among the first studies to combine detailed OCT morphometric analysis, microperimetric functional assessment, and explainable machine learning in patients undergoing inverted ILM flap surgery for full-thickness macular holes. Such an approach may contribute to the development of more accurate prognostic models and facilitate personalized preoperative counseling by providing a deeper understanding of the structural determinants of postoperative visual rehabilitation.

## 5. Conclusions

In conclusion, pars plana vitrectomy with the inverted ILM flap technique shows excellent anatomical closure and important functional recovery in eyes with full-thickness macular hole. Both structural restoration and improvement in central retinal sensitivity support an important relationship between structure and function. Preoperative macular hole geometry, especially minimum linear diameter and hole height, were the most important predictors of postoperative outcomes, with significant prognostic value beyond visual acuity alone. These findings show the importance of detailed OCT-based assessment in predicting surgical results and guiding clinical decision-making.

## Figures and Tables

**Table 1:** Demographic distribution, visual acuity, and macular hole characteristics.

Characteristics	
Age (years), median (IQR)	70 (67–73)
Sex, *n* (%)	
Male	16 (45)
Female	19 (55)
BCVA (LogMAR), mean (SD)	0.92 (0.29)
BCVA healthy eye (LogMAR), mean (SD)	0.28 (0.38)
Follow-up (days), median (IQR)	171.1 (90–259)
Macular hole characteristics, *n* (%)	
Stage (Gass classification)	
Stage 2	10 (28.6)
Stage 3	22 (62.9)
Stage 4	3 (8.6)
IVTS Classification	
Small	8 (23)
Medium	5 (14)
Large	22 (63)
H (µm), mean (SD)	432.4 (89.8)
BD (µm), mean (SD)	956.14 (367.14)
MLD (µm), mean (SD)	463.68 (229.83)
DHI, mean (SD)	0.47 (0.13)
MHI, mean (SD)	0.55 (0.35)
THI, mean (SD)	1.28 (0.98)
HFF, mean (SD)	64.55 (28.64)

BCVA, Best Corrected Visual Acuity; LogMAR, Logarithm of the Minimum Angle of Resolution; *n*, number of subjects or observations; H, height of the macular hole; BD, base diameter; MLD, minimum linear diameter; DHI, Diameter Hole Index; MHI, Macular Hole Index; THI, Tractional Hole Index; HFF, Hole Form Factor; IQR interquartile range (25–75%); SD, standard deviation.

**Table 2:** Functional and structural outcome at 2 follow-ups (1-month postoperative and final follow-up).

	Follow-Up 1	Follow-Up Final
Visual acuity, mean (SD)	0.69 (0.34)	0.70 (0.37)
Microperimetry, mean (SD)		
CMS (dB)	14.94 (9.50)	16.50 (9.50)
MS 4° (dB)	20.44 (4.36)	21.36 (4.47)
MS 20° (dB)	22.24 (3.17)	21.08 (3.91)
Microperimetry contralateral, mean (SD)		
CMS (dB)	19.31 (8.81)	NA
MS 4° (dB)	23.64 (4.47)	NA
MS 20° (dB)	24.18 (3.22)	NA
OCT		
ELM status, *n* (%)		*
Continuous	11 (31)	23 (66)
Discontinuous	9 (26)	6 (17)
Absent	15 (42)	6 (17)
ELM disruption diameter (µm), mean (SD)	842.29 (591.28)	672.25 (443.44) *
EZ status, *n* (%)		*
Continuous	2 (6)	14 (40)
Discontinuous	12 (34)	7 (20)
Absent	21 (60)	14 (40)
EZ disruption diameter (µm), mean (SD)	1320.81 (821.43)	1090.92 (766.16) *

CMS, Central Macular Sensitivity; MS, Mean Sensitivity; 4°, central 4-degree retinal area; 20°, central 20-degree retinal area; OCT, Optical Coherence Tomography; ELM, External Limiting Membrane; EZ, Ellipsoid Zone; SD, standard deviation; NA, not available. * Statistical significance, *p* < 0.05, comparison between the 2 follow-up points (Wilcoxon signed-rank for categorical variables, and *t*-test for continuous variables).

**Table 3:** Correlations between BCVA postoperative, microperimetry, and OCT parameters.

	Follow-Up 1 Spearman’s ρ	*p*-Value	Follow-Up Final Spearman’s ρ	*p*-Value
BCVA postoperative, and				
MH stage	**0.480**	**0.000**	**0.498**	**0.013**
OCT preoperative				
H	0.294	0.086	0.384	0.064
BD	0.600 **	**0.000**	0.706 **	**0.000**
MLD	0.580 **	**0.000**	0.612 **	**0.001**
DHI	0.156	0.371	0.146	0.497
MHI	−0.519 **	**0.001**	−0.574 **	**0.003**
THI	**−0.456**	**0.005**	−0.522 **	**0.009**
HFF	0.545 **	**0.001**	0.628 **	**0.001**
OCT postoperative				
ELM status	**0.498**	**0.002**	0.641 **	**0.033**
ELM disruption	0.375	0.071	0.577	0.308
EZ status	**0.412**	**0.014**	0.501 **	**0.013**
EZ disruption	**0.492**	**0.004**	0.433	0.244
Microperimetry CMS, and				
MH stage	−0.575 **	**0.000**	**−0.487**	**0.029**
OCT preoperative				
H	−0.251	0.145	−0.381	0.098
BD	**−0.477**	**0.004**	−0.687 **	**0.004**
MLD	−0.564 **	**0.000**	−0.672 **	**0.001**
DHI	−0.309	0.071	−0.150	0.529
MHI	**0.460**	**0.005**	0.564 **	**0.010**
THI	**0.471**	**0.004**	0.571 **	**0.009**
HFF	**−0.477**	**0.004**	−0.587 **	**0.006**
OCT postoperative				
ELM status	**−0.453**	**0.006**	−0.564 **	**0.010**
ELM disruption	−0.376	0.070	−0.264	0.341
EZ status	**−0.424**	**0.011**	**−0.467**	**0.038**
EZ disruption	−0.519 **	**0.002**	**−0.494**	**0.037**
Microperimetry 4°, and				
MH stage	−0.590 **	**0.000**	−0.549 **	**0.012**
OCT preoperative				
H	−0.085	0.628	−0.334	0.150
BD	**−0.408**	**0.015**	−0.693 **	**0.001**
MLD	−0.601 **	**0.000**	−0.697 **	**0.001**
DHI	**−0.515**	**0.002**	−0.245	0.298
MHI	0.518 **	**0.001**	0.610 **	**0.004**
THI	0.581 **	**0.001**	0.663 **	**0.004**
HFF	−0.511 **	**0.002**	−0.534 **	**0.015**
OCT postoperative				
ELM status	**−0.414**	**0.013**	**−0.472**	**0.015**
ELM disruption	−0.524 **	**0.009**	−0.628	0.070
EZ status	−0.231	0.181	−0.417	0.068
EZ disruption	−0.625 **	**0.000**	−0.553 **	**0.026**
Microperimetry 20°, and				
MH stage	**−0.405**	**0.016**	−0.360	0.119
OCT preoperative				
H	0.069	0.695	−0.249	0.290
BD	−0.193	0.266	−0.500 **	**0.025**
MLD	**−0.407**	**0.015**	**−0.487**	**0.029**
DHI	**−0.442**	**0.008**	−0,249	0.289
MHI	**0.383**	**0.023**	0.404	0.077
THI	**0.427**	**0.011**	0.409	0.074
HFF	**−0.440**	**0.008**	−0.566 **	**0.009**
OCT postoperative				
ELM status	**−0.409**	**0.015**	−0.314	0.178
ELM disruption	−0.387	0.062	−0.586	0.097
EZ status	−0.151	0.386	−0.132	0.579
EZ disruption	−0.502 **	**0.003**	−0.446	0.083

BCVA, Best Corrected Visual Acuity; MH, Macular Hole; OCT, Optical Coherence Tomography; H, height of the macular hole; BD, base diameter; MLD, minimum linear diameter; DHI, Diameter Hole Index; MHI, Macular Hole Index; THI, Tractional Hole Index; HFF, Hole Form Factor; ELM, External Limiting Membrane; EZ, Ellipsoid Zone; CMS, Central Macular Sensitivity; 4°, central 4-degree retinal area; 20°, central 20-degree retinal area; Statistic significance *p* < 0.05 in bold Bolded 1 more result (ELM status postoperative); ** Strong correlation.

**Table 4:** Comparison of prognostic models for postoperative central 4° microperimetry based on preoperative visual acuity and OCT-derived parameters.

Model	Variables		RMSE	R^2^
BCVA only		linear	4.2	0.042
Raw OCT only	H, MLD, BD	XGBoost	3.0	0.524
Indices only	THI, DHI, MHI	XGBoost	3.3	0.406
HFF		linear	3.8	0.219
BCVA + Raw OCT	BCVA, H, MLD, BD	XGBoost	1.7	0.850 **
BCVA + Indices	BCVA, THI, DHI, MHI	XGBoost	2.7	0.601
BCVA + All OCT	BCVA, H, MLD, BD, THI, DHI, MHI	XGBoost	2.4	0.700
BCVA + HFF		XGBoost	2.7	0.597

BCVA, preoperatory visual acuity; H, hole height; MLD, minimum linear diameter; BD, basal diameter; THI = H/MLD; DHI = MLD/BD; MHI = H/BD; HFF = 2 × sqrt(h^2^ + (BD−MLD)^2^)/BD. Models were constructed using linear regression or gradient boosting. The BCVA-only model was fitted using linear regression, as it included a single predictor. All other models were fitted using XGBoost to allow for nonlinear effects and interactions among preoperative OCT-derived variables. Model performance is reported using root mean squared error (RMSE) and coefficient of determination (R^2^). ** strongest predictor.

**Table 5:** Relative importance of preoperative raw OCT-derived variables in the XGBoost model predicting postoperative central 4° microperimetry.

Feature	Rank (Low–Better)	Gain
Hole height	1	0.328
Basal diameter	2	0.298
Minimum linear diameter	3	0.298
Preoperative BCVA	4	0.076

Variable importance is expressed as Gain, representing how much each variable contributes (relative contribution) to predict central microperimetry (model performance) in the XGBoost model. Gain values reflect predictive contribution and should not be interpreted as causal effects.

## Data Availability

The data presented in this study are available on request from the corresponding author due to privacy, legal or ethical reasons.

## Abbreviations

The following abbreviations are used in this manuscript:

BCVA	Best Corrected Visual Acuity
BD	base diameter
CMS	Central Macular Sensitivity
DHI	Diameter Hole Index
ELM	External Limiting Membrane
EZ	Ellipsoid Zone
FTMH	Full thickness macular hole
H	height of the macular hole
HFF	Hole Form Factor
ILM	Internal limiting membrane
IZ	Interdigitation zone
LogMAR	Logarithm of the Minimum Angle of Resolution
MH	Macular Hole
MHI	Macular Hole Index
MLD	minimum linear diameter
MS	Mean Sensitivity
OCT	Optical Coherence Tomography
SD	Standard Deviation
SHAP	Shapley Additive Explanations
THI	Tractional Hole Index
